# Aseptic abscess syndrome in an adolescent with congenital heart defect and multiorgan inflammation: a case report

**DOI:** 10.1016/j.ero.2025.12.013

**Published:** 2026-01-10

**Authors:** Olga B Krammer, Philipp Caspritz, Vlad Pavel, Stephan Schmid, Martina Müller, Martin Fleck

**Affiliations:** 1 Department of Rheumatology and Clinical Immunology, Asklepios Hospital Bad Abbach, Bad Abbach, Germany; 2 Department of Internal Medicine I, University Medical Center, Regensburg, Germany

## Abstract

We report the case of a 19-year-old male patient with recurrent fever and multiple sterile abscesses involving lymph nodes, subcutaneous tissue, and joints, associated with a congenital cardiac anomaly. The patient presented with persistent febrile episodes and systemic inflammation despite broad-spectrum antibiotic therapy. Imaging revealed disseminated lymphadenopathy and abscesses in cervical, subpectoral, mesenteric, and articular regions. Extensive microbiologic and serologic workup, including bacterial, mycobacterial, and viral testing, remained negative. Histopathology showed necrotising and suppurative lymphadenitis without identifiable pathogens. A diagnosis of aseptic abscess syndrome was established. Treatment with high-dose prednisolone followed by methotrexate led to rapid clinical and laboratory improvement, with normalisation of inflammatory markers and no recurrence of abscesses. This case highlights the importance of recognising aseptic abscess syndrome as a differential diagnosis in patients with culture-negative abscesses and systemic inflammation, to ensure timely immunosuppressive treatment and avoid unnecessary antimicrobial therapy.

## INTRODUCTION

Aseptic abscess syndrome (AAS) is a rare disorder characterised by deep, sterile, neutrophil-rich abscesses that often mimic bacterial infection or malignancy. The condition predominantly affects young adults and can involve multiple organ systems. Because clinical and radiologic findings resemble infection, diagnosis is frequently delayed until microbiologic studies remain persistently negative. Early recognition is crucial, as AAS typically responds to corticosteroids or immunomodulatory therapy, whereas antibiotic treatment is ineffective.

## CASE REPORT

In May 2025, a 19-year-old male patient was admitted to a peripheral hospital for recurrent febrile episodes up to 39°C. He denied dyspnoea, dysuria, or other genitourinary symptoms. There was no family history of heart disease or congenital disease, no recent travel, and no relevant exposure or sexual history.

From December 2024 to April 2025, he experienced episodes of diarrhoea and abdominal pain. Extensive gastrointestinal evaluation was unremarkable: stool testing was negative for *Clostridioides difficile, Campylobacter, Yersinia, Shigella*, and *Salmonella*. Duodenal biopsy showed no evidence of coeliac disease, Morbus Whipple, or Giardiasis. However, ileocolonoscopy revealed mild terminal ileitis. Calprotectin testing in stool was not performed.

**Figure 1:**
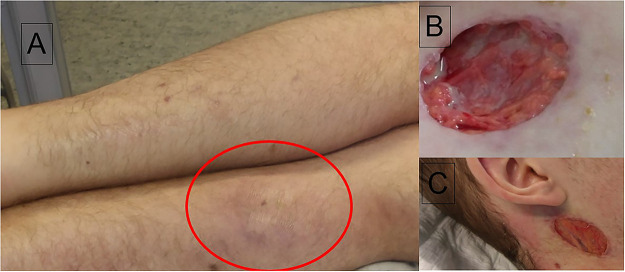
Clinical Images of abscesses. A, Dorsum of the right lower leg. B, Left clavicular. C, Right cervical region.

Laboratory evaluation revealed markedly elevated inflammatory parameters (C-reactive protein [CRP] up to 290 mg/L, leukocytes 28/nL, and procalcitonin 0.26 ng/mL). Pneumococcal and Legionella urinary antigens, as well as a mononucleosis screening test, were negative. Blood and sputum cultures remained sterile. Thoracic imaging showed no signs of pneumonia. Abdominal ultrasound and magnetic resonance imaging (MRI) demonstrated mesenteric lymphadenopathy with bulky, partly necrotic, and cystic lymph nodes. Endoscopic gastrointestinal evaluation was otherwise unremarkable. Cardiac imaging revealed a previously undiagnosed congenital atrial septal defect of the sinus venosus type with anomalous pulmonary venous drainage into the right atrium, resulting in a right-to-left shunt and pulmonary arterial hypertension. Serial blood cultures were repeatedly negative. In the clinical course, multiple erythematous nodular lesions (cervical, pectoral, and frontal) developed (Fig 1). Surgical drainage of the cervical lesion yielded no bacterial growth, and subsequent wound cultures and broad-range polymerase chain reaction (PCR) assays (bacterial, mycobacterial, fungal) were negative. Autoimmune testing (antinuclear antibody, extractable nuclear antigen, complement, and immunoglobulins) and cerebrospinal fluid studies were unremarkable. Despite treatment with meropenem, fever and inflammatory markers persisted.

**Figure 2:**
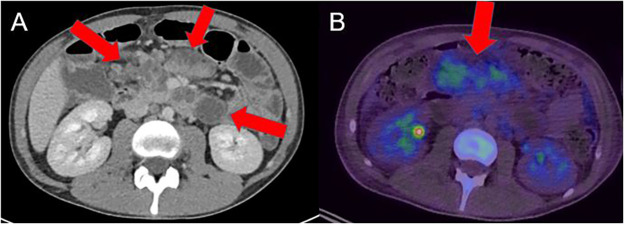
A, Contrast-enhanced abdominal CT showing conglomerated lymph nodes consistent with bulky disease. B, PET/CT demonstrating FDG uptake in multiple mesenteric lymph nodes in the abdominal region. CT, computed tomography; FDG, fluorodeoxyglucose; PET, positron emission tomography.

In June 2025, the patient was transferred to the University Hospital Regensburg for further evaluation due to prominent bulky abdominal lymphadenopathy and suspected mesenteric cystic lymphangioma (Fig 2). Laboratory studies confirmed elevated CRP, mild procalcitonin elevation, and neutrophilia with normal renal parameters. Serologic and PCR testing for HIV, cytomegalovirus, Epstein–Barr virus, Brucella, Bartonella, Toxoplasma, syphilis, Echinococcus, and tuberculosis were negative. Autoantibody screening (antinuclear antibody and antineutrophil cytoplasmic antibody) was within normal limits. Computed tomography (CT) and fluorodeoxyglucose (FDG)-positron emission tomography (PET)/CT imaging demonstrated widespread FDG-avid lymphadenopathy (punctum maximum mesenterial), FDG-positive synovitis of multiple joints, and multiple subcutaneous inflammatory lesions (Fig 2). As PET-CT demonstrated increased bone marrow uptake, a bone marrow aspiration was performed, without signs of malignancy or infectious origin. A transesophageal echocardiography showed no evidence of endocardial vegetations. An MRI of the cervical spine showed inflammatory enhancement around the dens axis, consistent with early spondylitis.

Histopathologic evaluation of multiple biopsies (abdominal and inguinal lymph nodes) revealed necrotising and suppurative lymphadenitis without evidence of malignancy or microorganisms. Skin biopsy showed an acute neutrophil-rich necrotising inflammation. Microbiologic cultures and PCR assays for *Mycobacterium* spp, Leishmania, human herpesvirus 8, and fungi remained negative. The arthritis and abscess formation in the wrist and metatarsophalangeal joints were surgically drained with negative cultures.

Despite broad antimicrobial therapy with meropenem, doxycycline, metronidazole, and vancomycin, inflammatory markers continued to rise (max. CRP 390 mg/L and leukocytes 37/nL). The patient experienced intense pain in the abdominal regions as well as in the joints and skin, requiring escalation of intravenous analgesic therapy to sufentanil.

**Figure 3:**
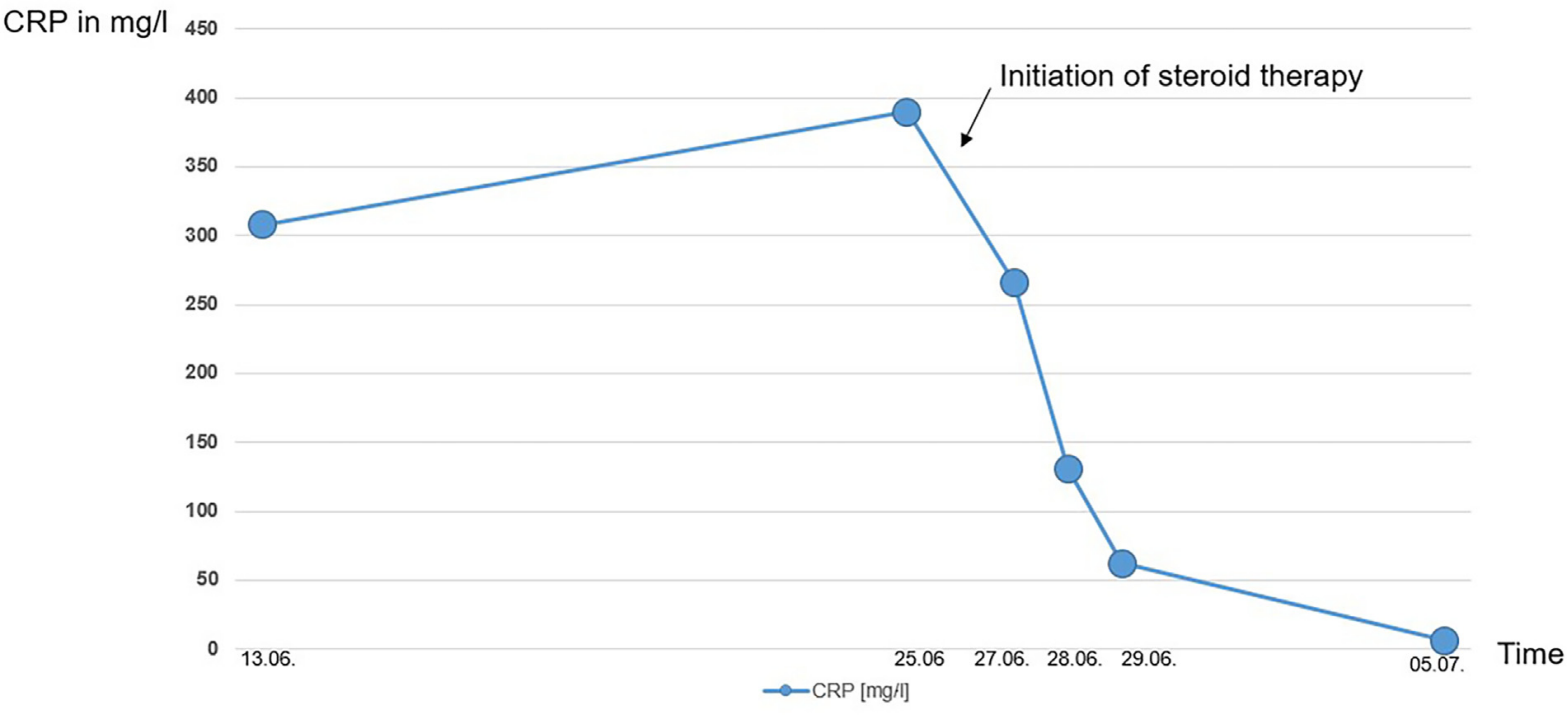
Rapid improvement of systemic inflammation following initiation of corticosteroid therapy.

Given the sterile abscesses and negative microbiology, the AAS was suspected. A continuous hydrocortisone infusion (20 mg/h) was started. The systemic inflammation and pain rapidly improved, allowing tapering and discontinuation of antibiotics (Fig 3). Due to the negative microbiology, sterile abscesses, and the rapid steroid responsiveness, a diagnosis of AAS was established. An enteropathic spondyloarthritis was considered but deemed unlikely, as HLA-B27 testing was negative and MRI of the lower spine and sacroiliac joints revealed no abnormalities. However, given the history of terminal ileitis and prior diarrheal episodes, an early or evolving inflammatory bowel disease (IBD) could not be excluded, as AAS has been described as a potential prodromal manifestation of Crohn disease [1].

The therapy was switched from continuous hydrocortisone infusion to oral prednisolone at 1 mg/kg/d, with subsequent tapering according to clinical response. Methotrexate 15 mg subcutaneously once weekly was initiated, and the treatment was well tolerated. Inflammatory markers normalised, and no new abscesses developed. Continued rheumatologic and cardiologic follow-up was recommended, given the congenital heart defect and systemic inflammatory condition.

## DISCUSSION

AAS is a rare inflammatory condition characterised by sterile, neutrophil-dominant abscesses in lymphoid and parenchymal tissues. It can mimic infectious and malignant diseases, often leading to a diagnostic delay. By many investigators, it is considered to be a visceral variant of neutrophilic dermatoses (such as pyoderma gangrenosum or Sweet Syndrome). AAS is also grouped among autoinflammatory disorders [1,2]. The pathophysiology is incompletely understood but involves dysregulated neutrophil activation and cytokine-driven inflammation. To date, no specific biomarker has been established [2,3].

AAS can occur as an isolated idiopathic condition or in association with systemic diseases such as IBD. In the largest cohort reported from France (*n* = 71) and in published cases outside France, IBD was present in 31.5% and 42.2% of patients, with AAS preceding the diagnosis of IBD in approximately 20%, highlighting its potential role as an early systemic manifestation [3,4]. Recognising this relationship is crucial, as gastrointestinal manifestations may appear months or even years after the first episode of aseptic abscess formation. In the present case, the patient’s history of diarrhoea and terminal ileitis raises the possibility of an evolving IBD phenotype, warranting longitudinal follow-up. Neutrophilic dermatoses were frequently reported, with pyoderma gangraenosum observed in 14.3% of patients in the French cohort and in 37% of cases from international series. In contrast, associations with inflammatory rheumatic diseases were uncommon: spondyloarthritis was documented in 4.2% of patients in the French cohort, whereas rheumatoid arthritis was reported in 1.4% of French cases and 5.6% of cases outside France. Overall, these findings suggest that the observed overlap reflects shared neutrophil-dependent pathogenic mechanisms rather than a strong epidemiological association [3,4].

Although AAS has clinical and mechanistic overlap with PSTPIP1-associated syndromes (especially PAPA), it is not formally part of this spectrum. In contrast to these predominantly monogenic disorders, AAS is regarded as a polygenic or multifactorial autoinflammatory disease without a consistent causative mutation [5]. Shared features include sterile neutrophil-rich inflammation and reported responses to interleukin 1 blockade. Phenotypically, AAS differs by predominantly manifesting as visceral sterile abscesses rather than the classic skin–joint involvement characteristic of PAPA-spectrum diseases. Corticosteroids remain the mainstay of therapy and typically lead to rapid remission of fever and abscesses. In steroid-dependent, relapsing, or nonresponsive disease, immunomodulatory therapies are required. Methotrexate and azathioprine are commonly used steroid-sparing agents, whereas TNF-*α* inhibitors, particularly infliximab and adalimumab, are highly effective in refractory cases, especially when associated with IBD. In isolated refractory presentations, interleukin 1 blockade with anakinra or canakinumab has shown benefit in case reports [3,4]. Early recognition and immunosuppressive treatment are key to preventing unnecessary antibiotic exposure and to controlling systemic inflammation [4].

This case highlights the diagnostic challenges of AAS in adolescents with systemic inflammation and negative microbiologic studies. The presence of a congenital cardiac anomaly further complicated the evaluation due to overlapping inflammatory and infectious differentials. Nevertheless, prompt initiation of corticosteroid therapy led to full remission, underscoring the importance of considering AAS in the differential diagnosis of culture-negative abscesses, especially when gastrointestinal symptoms or terminal ileitis suggest a potential link to IBD [3].

## Funding

This research did not receive any specific grant from funding agencies in the public, commercial, or not-for-profit sectors.

## CRediT authorship contribution statement

**Olga B Krammer:** Writing – original draft, Visualization, Conceptualization. **Philipp Caspritz:** Writing – review & editing. **Vlad Pavel:** Writing – review & editing, Resources. **Stephan Schmid:** Writing – review & editing. **Martina Müller:** Validation. **Martin Fleck:** Writing – review & editing, Supervision.

## Competing interests

All authors declare they have no competing interests.
